# MARKET DAYS: INTERGENERATIONAL PROGRAMMING TO SUPPORT HEALTHY AGING AMONG LOW-INCOME OLDER ADULTS

**DOI:** 10.1093/geroni/igae098.1640

**Published:** 2024-12-31

**Authors:** Allyson Brothers, India Luxton, Cheryl Noble, Christa Timmerman

**Affiliations:** Colorado State University, Fort Collins, Colorado, United States; Colorado State University, Fort Collins, Colorado, United States; Larimer County CSU Extension, Fort Collins, Colorado, United States; Larimer County CSU Extension, Fort Collins, Colorado, United States

## Abstract

The Market Days for Older Adults program provides fresh produce to food-insecure older adults at the downtown farmers’ Market. As part of an ongoing university-community service partnership, undergraduate internships and service-learning projects enhance older adults’ social connections while training gerontology students about health disparities and aging. The program addresses two Age-Friendly University (AFU) principles: #4: to promote intergenerational learning; and #6: to ensure the research agenda is shaped by needs of older adults. A 2023 program evaluation of Market Days (MD) in 2023 employed a pre-post mixed methods design (N=59) to examine process and outcome variables. We found evidence for high satisfaction and usage of the program, plus multiple perceived benefits to participants, including improved sense of health (61%), new ways for preparing fresh produce (31%), and higher connection to the community. We did not find evidence of significant improvements in food insecurity or social isolation. Students reported an increased understanding of the role of community programs in promoting healthy aging, and appreciation of food insecurity as a risk factor for aging. Results indicated that this subpopulation of older adults is particularly vulnerable with regard to chronic illness, low preparations for aging, low community resource awareness, mental health symptoms, and negative views on aging. The results will inform future steps of our community-engaged research program. Viewing social isolation as a pressing social determinant of health in low-income older adults, we will develop a formal intergenerational programming component to further strengthen social connections, while also enhancing undergraduate gerontology student training opportunities.

